# Trends in mortality rates of female breast cancer in China, 2004 to 2021

**DOI:** 10.1097/MD.0000000000046805

**Published:** 2026-01-16

**Authors:** Huali Xiong, Fengxun Ma

**Affiliations:** a Department of Chronic Noncommunicable Disease Prevention and Control, Center for Disease Control and Prevention of Rongchang District, Chongqing, China; b Department of Public Health, The People’s Hospital of Rongchang District, Chongqing, China.

**Keywords:** breast cancer, female, joinpoint, mortality rate, trends

## Abstract

National-wide estimates of female breast cancer (BC) mortality rates in China are largely derived from the global burden of disease study or global cancer statistics, population-based surveillance data remain scarce. The current study aims to analyze the trend changes of female BC mortality rates from 2004 to 2021 and to inform targeted control strategies. Age-specific deaths and mortality rates from 2004 to 2021 were extracted from the Chinese Cause-of-Death Surveillance Dataset. Joinpoint regression model was applied to explore the trend changes with average annual percent change (AAPC) in mortality rates. A total of 108,063 occurred due to female BC from 2004 to 2021. The crude mortality rate (CMR) (AAPC = 2.21%) showed an increased trend and the age standard mortality rate (AAPC_China_ = 0.68%, AAPC_world_ = −0.29%) remained relatively stable from 2004 to 2021. Despite higher CMR in urban areas, the absolute urban deaths were fewer. Both urban and rural areas experienced an upward trend in CMR (AAPC_urban_ = 1.08%, AAPC_rural_ = 3.13%). The age standard mortality rate in urban and rural areas remained relatively stable (AAPC_urban_ = −0.13%, AAPC_rual_ = 1.35%). Age-stratified analysis showed the CMR remained relatively stable in females, whereas urban females at age of 0 to 24 years (AAPC = 26.98%) and rural females exhibited significant increases at ages of 25 to 34 years (AAPC = 2.31%), 35 to 64 years (AAPC = 0.80%), ≥65 years (AAPC = 1.01%). Expanding screening coverage in rural areas and improving the accessibility of BC screening and treatment for the elderly can effectively enhance the utilization of healthcare resources.

## 1. Introduction

Breast cancer (BC) is the leading cause of morbidity and mortality among females globally.^[1]^ The Global cancer statistics 2022 indicated that there were approximately 2.3 million new cases of BC, accounting for 11.60% of all malignant tumors in females, with the incidence ranking first among females.^[2]^ There were approximately 666,000 deaths due to BC, representing 6.90% of all malignant tumor deaths among females.^[2]^ The high mortality rates were primarily concentrated in less developed countries, such as Jamaica, the Bahamas, Barbados, Samoa, Cameroon, Nigeria and especially Fiji, which had the highest mortality rate of 38.9/100,000.^[3]^ In 2021, there were 20.26 million disability-adjusted life years due to female BC.^[4]^ By 2050, deaths are projected to reach 1.48 million among females. To counter the threats and challenges posed by females BC worldwide, the World Health Organization launched the Global BC Initiative, aiming to reduce the mortality rate of BC by 2.50% annually through the promotion of health education, early detection, and comprehensive treatment.^[5,6]^

BC also represents a major public health concern in China. The age-standardized mortality rate (ASMR) of female BC in China was relatively low, ranking 181st out of 185 countries.^[7]^ However, the mortality rate of BC had increased rapidly over the last 2 decades^[8]^ in China. According to the Chinese cancer registration data (edition 2018), BC was the second most commonly diagnosed malignancy and the fifth leading cause of cancer-related death among Chinese females.^[9]^ Moreover, the disability-adjusted life years attributabled to female BC demonstrated a consistent increase from 1990 to 2019.^[10]^ It is predicted that the annual number of BC deaths in China will exceed 100,000 cases by 2030.^[11]^ Consequently, establishing targeted intervention measures for the prevention and control of BC is crucial.

Currently, studies on females BC mortality in China were primarily based on the Global Disease Burden^[10–12]^ or Global cancer statistics.^[7]^ However, comprehensive analysis of the recent mortality trends of female BC based on real-world, national surveillance data is lacking. To address this knowledge gap, the current study utilized the most recent cause-of-death surveillance data to explore the national mortality trend changes of female BC in China. Specifically, we applied Joinpoint regression model to analyze the temporal trends in female BC mortality from 2004 to 2021. These findings are expected to provide a scientific basis for the government to update BC prevention and control strategies and to inform evidence-based public health policies.

## 2. Data and methods

### 2.1. Data sources

The classification code for female BC is C50,^[13]^ according to the International Statistical Classification of Diseases and Related Health Problems, 10th Revision (ICD-10). Data on deaths, crude mortality rate (CMR), age-standardized mortality rate (ASMR) classified by area (urban or rural) and age group due to female BC, as well as the population of monitored areas were extracted from the China cause-of-death disease surveillance datasets from 2004 to 2021 (https://ncncd.chinacdc.cn/jcysj/siyinjcx/syfxbg/). The classification of rural areas in China covers all counties and county-level cities, while urban areas were defined by districts. The age standard population in China was based on the Sixth National Census in 2010. The subgroups of age were divided into <24 years, 25 to 34 years, 35 to 64 years, and ≥65 years according to the age stratification used in Chinese BC research. Because the deaths from female BC in those under 24 years is small, the 34 to 65 years age group reflects a truncated mortality rate^[14]^ and age group over 65 represents the mortality rate of the elderly.

This study constituted a secondary analysis of surveillance data extracted from the Chinese Cause-of-Death Surveillance Datasets from 2004 to 2021. The datasets contained no personal identifiers (name, ID number, address, or telephone) and were published. the need for formal ethics review was waived by the Ethics Committee of Center for Disease Control and Prevention of Rongchang District, because the study involved only publicly mandated surveillance information with no additional contact with living individuals.

The development of the Chinese cause-of-death surveillance system has gone through 3 time periods. The first period was from 1978 to 1989. The National Disease Surveillance Points (DSP) were officially established in Dongcheng District and Tongzhou District of Beijing in 1978 and grew to 71 monitoring sites by 1989, covering 29 provinces. The second period was from 1990 to 2002. Using a multi-stage stratified cluster random sampling method, representative DSP were chosen from all 31 provinces, forming a new DSP system with 145 monitoring points. This system covered a monitored population of 10 million, accounting for about 1% of China’s total population. The third period was from 2003 to 2012. During this period, The DSP system was modified, increasing the number of DSP to 161 across all 31 provinces, covering a monitored population of over 77 million, which represents about 6% of the country’s population. Since 2013, the Chinese government had merged and broadened the original Ministry of Health’s cause-of-death statistical system, the National Disease Surveillance System, and other death reporting systems to create a nationally representative system. After the integration, the number of monitoring points has increased to 605, with a monitored population exceeding 300 million, covering approximately 24% of the national population. The cause-of-death surveillance data for the years 2001 to 2003 were not published, and the latest surveillance data were available up to 2021. Consequently, the timeline from 2004 to 2021 was selected to analyze the trends in mortality rates for female BC.

### 2.2. Quality control

In the current study, the data extraction was performed independently by 2 medical professionals with the title of attending physician and associate chief physician. Based on the extracted deaths and corresponding population data from the same surveillance datasets, the CMR and ASMR were recalculated. These recalculated rates were then systematically compared against the originally extracted CMR and ASMR values. If any inconsistencies were found, immediate reexamination and modification were carried out. The data extraction process was considered complete and the data deemed ready for analysis only after the recalculated mortality incidence were verified to be fully consistent with those entered by the 2 medical professionals, as confirmed by a third professional through cross-referencing with the China cause-of-death disease surveillance datasets from 2004 to 2021.

### 2.3. Statistical analysis

The Joinpoint Regression Model is a statistical method used to analyze temporal trends in mortality rates of malignant tumors.^[15]^ The fundamental concept of Joinpoint regression analysis is to divide a long-term trend line into several statistically significant trend segments through model fitting, with each segment described by a continuous linear function. The points where different trend segments are referred to as joinpoints, and the number and statistical significance of joinpoints are determined using the Monte Carlo test.^[16,17]^ If the number of joinpoints is zero, the trend graph is a straight line; if the number of joinpoints is not zero, the trend is represented by several consecutive line segments. The Joinpoint regression equation is:


$$\begin{array}{l} \log(y|x)=\beta 0+\beta 1x+\delta 1{\left(x-\tau 1\right)}^{+}+\cdot \cdot \cdot \cdot \cdot \cdot +\delta k{\left(x-\tau k\right)}^{+} \end{array}$$

y refers to mortality rate (/100,000), χ refers to observation year, β refers to the constant term in the regression model, β1 refers to regression coefficients for each segment function (i = 1,2,3,…,k); δ refers toregression coefficients for each segment function (i = 1,2,3,…,k). τi refers to unknown joinpoints (i = 1,2,3,…,k); if${\left(\chi -\tau k\right)}^{+}>0$, ${\left(\chi -\tau k\right)}^{+}=\left(\chi -\tau k\right)$, otherwise, ${\left(\chi -\tau k\right)}^{+}=0$, k refers to the number of joinpoints to be determined.

The current study employed the Joinpoint regression model to analyze the trends in CMR and ASMRs, as well as the standardized mortality rates for urban and rural females from 2004 to 2021. The mortality rates were log-transformed and used as the dependent variable, with the year as the independent variable to establish a log-linear model with a Poisson distribution. The annual percent change (APC) and the average APC (AAPC) were calculated for each indicator. To describe the trend changes in mortality rates, APC and AAPC with 95% confidence intervals were utilized. The formulas for calculating APC^[15]^ and AAPC^[18]^ are as follows:


$$\begin{array}{l} log\left(y\;|\;\chi \right)=b0+b1x \end{array}$$

$$\begin{array}{l} APCi=(e^{bi}-1)\times 100\% \end{array}$$

$$\begin{array}{l} AAPC=(e^{\frac{\sum wibi}{\sum wi}}-1)\times 100\% \end{array}$$

y refers to mortality rate (/100,000), χ refers to observation year, b_0_ refers to the intercept term of the log-linear regression, b_i_ refers to the slope coefficients for each segment during the observation period, w_i_ refers to the length of each segment during the observation period. If the 95% CI of APC and AAPC includes zero, it indicates that the change is not statistically significant (*P*>.05),^[18]^ Otherwise, if the 95% CI of the APC and AAPC is different from zero, it is considered statistically significant. APC and AAPC were tested by *t*-test.

ASMR_China_ was calculated using the Sixth National Population Census (2010) and ASMR_world_ was calculated by Segi 1960 world standard population.^[19]^ The truncated mortality rate typically refers to the mortality rate for the population in the age group of 35 to 64 years.^[20]^ Data were organized using Excel 2016 and charts were created by GraphPad Prism 8.0 software. Joinpoint 4.0.4 software was applied to calculate the APC and AAPC. All tests were conducted as 2-sided, with a *P*-value of less than .05 deemed statistically significant.

## 3. Results

### 3.1. Overall death characteristic of due to female breast cancer

**Figure 1.**
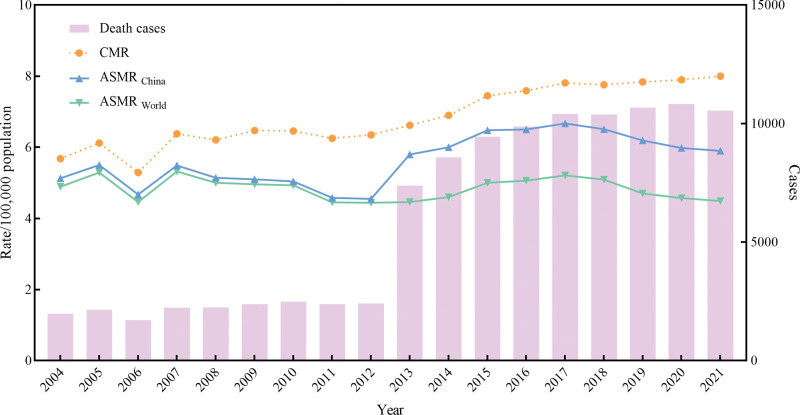
Overall death characteristic due to female breast cancer (BC) in China, 2004 to 2021. ASMR = age-standardized mortality rate, CMR = crude mortality rate.

The CMR, ASMR by Chinese standard population (ASMR_china_) or Segi 1960 world standard population (ASMR_world_) and deaths due to female BC were shown in Figure 1. A total of 108,063 cases were attributed to female BC from 2004 to 2021. The CMR was 5.68/100,000 population in 2004 and changed to 8.00/100,000 population in 2021. The ASMR_china_ and ASMR_world_ were 5.13/100,000 population, 4.89/100,000 population in 2004 and changed to 5.90/100,000 population, 4.49/100,000 population in 2021, respectively.

### 3.2. Trends of female breast cancer mortality

**Figure 2.**
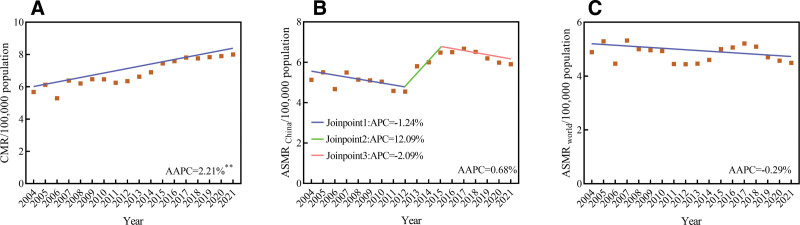
Trends in mortality rates of female breast cancer (BC) in China, 2004 to 2021. (A) CMR; (B) ASMR_China_; (C) ASMR_world_.. ** *P*<.05. AAPC = average annual percent change, APC = annual percent change, ASMR_China_ = age-standardized mortality rate by Chinese standard population, ASMR_world =_ age-standardized mortality rate by Segi 1960 world standard population, CMR = crude mortality rate.

The CMR showed an increased trend (AAPC = 2.21%, 95% CI: 1.77%–2.65%, *t* = 10.753, *P*<.001) from 2004 to 2021, with no segments identified. The ASMR_china_ and ASMR_world_ remained relatively stable from 2004 to 2021 (AAPC_China_ = 0.68%, 95% CI: −2.03% to 3.47%, *t* = 0.485, *P* = .627; AAPC_world_ = −0.29%, 95% CI: −0.90% to 0.33%, *t* = −0.979, *P* = .342) (Fig. 2).

### 3.3. Trends of female breast cancer mortality by areas

**Figure 3.**
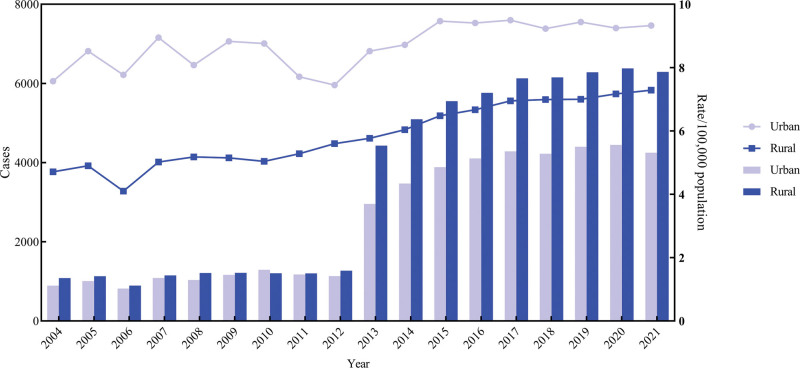
The crude mortality and death cases by areas in China, 2004 to 2021. CMR = crude mortality rate.

**Figure 4.**
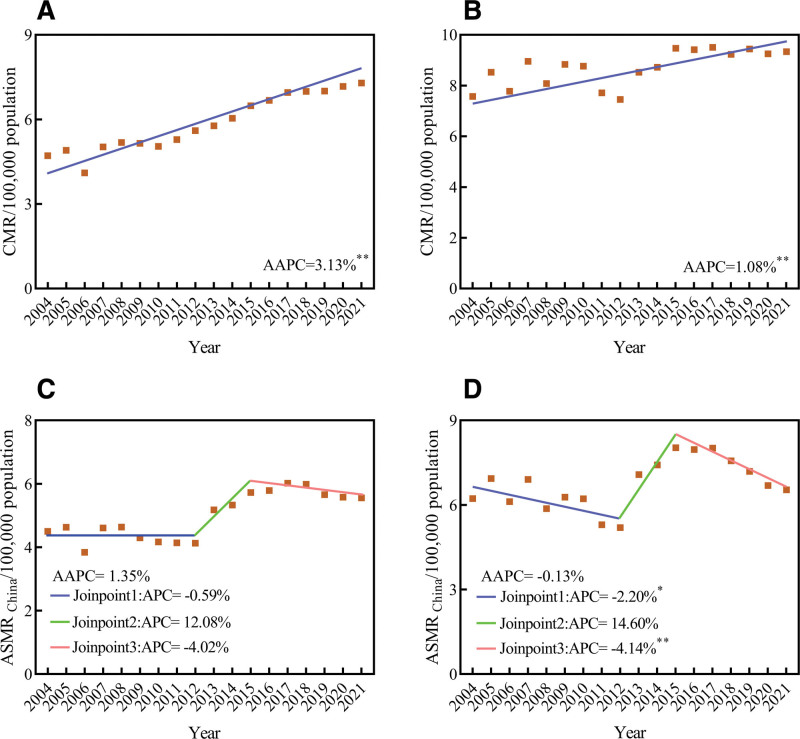
Trends in mortality rates of female BC in China stratified by urban/rural areas, 2004 to 2021. (A) CMR for rural areas; (B) CMR for urban areas; (C) ASMR_China_ for rural areas; (D) ASMR_China_ for urban areas. ASMR_China_ = age-standardized mortality rate by Chinese standard population, AAPC = average annual percent change, APC = annual percent change, CMR = crude mortality rate. ** *P* <.01; * *P* <.05.

From 2004 to 2021, despite the CMR was higher in urban areas compared to rural areas, the absolute deaths was lower in urban areas (Fig. 3). Both urban and rural areas experienced an upward trend in CMR (Urban: AAPC = 1.08%, 95% *CI*: 0.48%–1.69%, *t* = 3.831, *P* = .001; Rural: AAPC = 3.13%, 95% CI: 2.62%–3.64%, *t* = 13.205, *P*<.001). The ASMR_china_ in urban areas (AAPC = −0.13%, 95% CI: −3.28% to 3.12%, *t* = −0.080, *P* = .936) and rural areas (AAPC = 1.35%, 95% CI: −1.66%–4.45%, *t* = 0.871, *P* = .383) demonstrated to be maintained at a relatively stable level (Fig. 4).

### 3.4. Trends of female breast cancer mortality by age groups

**Figure 5.**
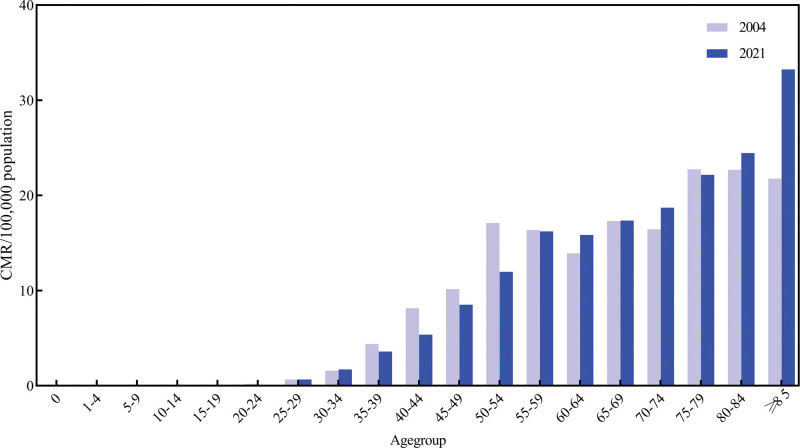
Female BC mortality rates between 2004 and 2021 stratified by age. CMR = crude mortality rate.

**Figure 6.**
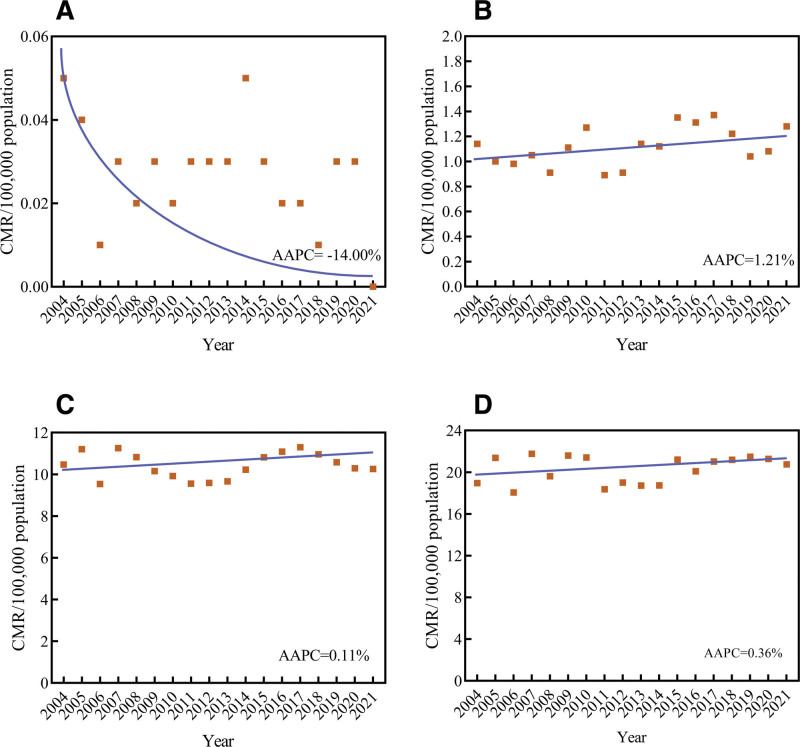
Trends in mortality rates of female BC in China stratified by age, 2004 to 2021. (A) CMR for 0 to 24 yr; (B) CMR for 25 to 34 yr; (C) CMR for 35 to 64 yr; (D) CMR for ≥65 yr. AAPC = average annual percent change, CMR = crude mortality rate.

**Figure 7.**
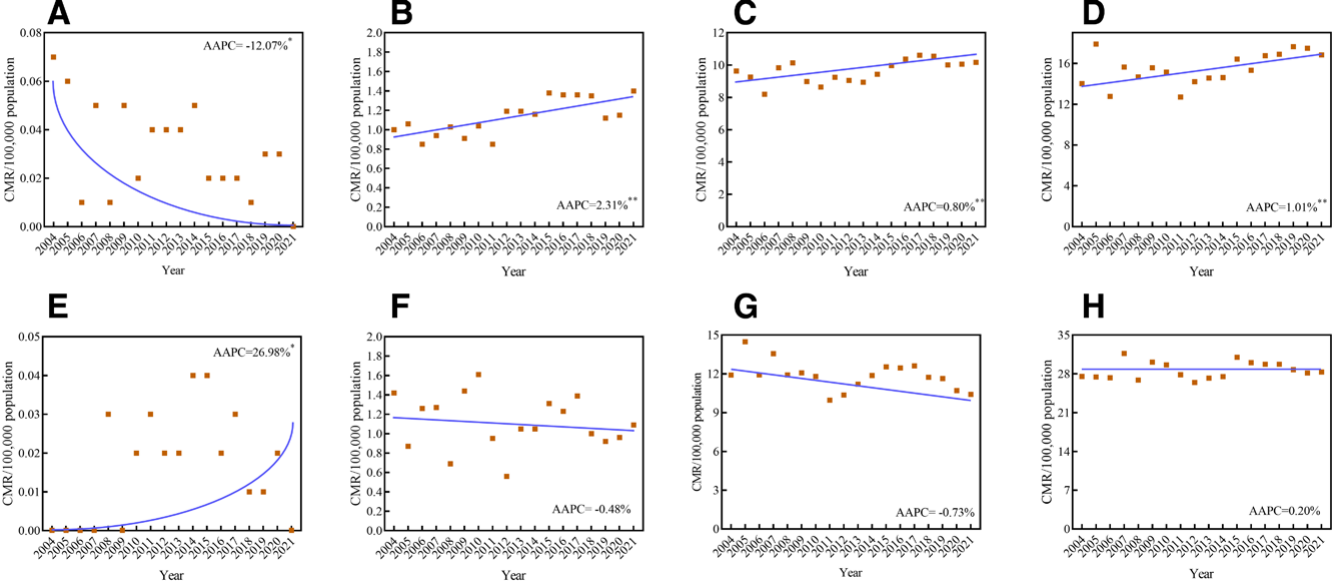
Trends in mortality rates of female BC in China stratified by age and areas, 2004 to 2021. (A) CMR for rural areas aged 0 to 24 yr; (B) CMR for rural areas aged 25 to 34 yr; (C) CMR for rural areas aged 35 to 64 yr; (D) CMR for rural areas aged ≥65 yr. (E) CMR for urban areas aged 0 to 24 yr; (F) CMR for urban areas aged 25 to 34 yr; (G) CMR for urban areas aged 35 to 64 yr; (H) CMR for urban areas aged ≥65 yr. AAPC = average annual percent change, CMR = crude mortality rate.

Age-stratified analysis indicated that mortality rates increased with age, with the majarity of deaths occurring in individuals aged 85 years or above (Fig. 5). The mortality trend for female BC exhibited a stable trend in the agegroup of <24 years (AAPC = −14.00%, 95% CI:−27.54% to 2.06%, *t* = −1.867, *P* = .080), 25 to 34 years (AAPC = 1.21%, 95% CI: −0.01% to 2.43%, *t* = 2.102, *P* = .051), 35 to 64 years (AAPC = 0.11%, 95% CI: −0.47% to 0.69%, *t* = 0.398, *P* = .695), and ≥65 years (AAPC = 0.37%, 95% CI: −0.25% to 0.97%, *t* = 1.356, *P* = .227) (Fig. 6). Additionally, further analysis was conducted to examine trends by age group in urban and rural areas, we found a downward in 25 to 34 years for rural areas (AAPC = −12.07%, 95% CI: −21.83% to −9.46%, *t* = 2.256, *P* = .042) (Fig. 7A) and upward trends in 25 to 34 years (AAPC = 2.31%, 95% CI: 1.83%–3.46%, *t* = 4.361, *P*<.001) (Fig. 7B), 35 to 64 years (AAPC = 0.80%, 95% CI: 0.21% to 1.39%, *t* = 2.895, *P* = .010) (Fig. 7C), ≥65 years (AAPC = 1.01%, 95% CI: 0.13%–1.90%, *t* = 2.437, *P* = .027) (Fig. 7D) in rural areas. On the contrary, we found an increased trend in 25 to 34 years (Fig. 7E) for urban areas (AAPC = 26.98%, 95% CI: 11.83%–33.46%, *t* = 3.256, *P* = .032), while the CMR in urban areas for the 25 to 34 years (Fig. 7F), 35 to 64 years (Fig. 7G), and ≥65 years (Fig. 7H) remained relatively stable.

## 4. Discussion

The present study was designed to examine the trends in female BC mortality rates in China from 2004 to 2021, with the aim of providing targeted interventions to improve the quality of life for patients with BC, and to offer scientific recommendations for the development of public health prevention and control strategies as well as the allocation of health resources. The CMR for female BC demonstrated a consistent upward trend from 2004 to 2021, which was consistent with previous studies based on GBD study.^[21,22]^ This increase can be partly attributed to well-established risk factors, such as tobacco use,^[4,23]^ diet high in red meat,^[21]^ high body mass index,^[24–26]^ low physical activity,^[27]^ alcohol use and secondhand smoke.^[28–31]^ Our study indicated that the inflection point in female BC deaths was 2013. The shift could be attributed to the use of a revised surveillance disease point as part of the national cause-of-death surveillance system initiated in 2013. Overall, the CMR of females BC showed an upward trend (AAPC = 2.21%, *P*<.05), whereas the ASMR remained relatively stable (AAPC = 0.68%, *P*>.05). This discrepancy may be explained by the increasing aging population and declining birth rate in China. Demographic data indicated that the population aged ≥60 years in China increased by nearly 150 million from 1990 to 2019, while the birth rate dropped from 21.06‰ in 1990 to 10.48‰ in 2019.^[32]^

The current study revealed that both the CMR and ASMR for female BC were greater in urban regions compared to rural regions, which was aligning with previous findings in Israel^[33]^ and the United States.^[34]^ This urban-rural disparity in mortality may be attributed to a higher incidence in urban populations, potentially driven by reproductive factors such as prolonged menstruation, fewer pregnancies, later age at first childbirth, and lack of breastfeeding among urban females.^[35]^ Furthermore, urban females were more likely to adopt adverse and unhealthy lifestyle, such as the increased consumption of high calorie foods and alcohol. Additionally, mental stress, air pollution, overweight and obesity were also contributed to a higher risk of BC incidence and mortality among urban females.^[36,37]^ Conversely, the steeper increasing trend in CMR observed in rural areas was a significant concern, which may be attributed to factors, including the relatively lower educational level among rural females, limited awareness of actively undergoing BC screening, unequal access to health care, and the unequal distribution of medical and health resources between urban and rural areas.^[38]^ Consequently, a majority of rural females were diagnosed with BC in middle or late stages, leading to poorer survival outcomes.^[39]^ In contrast, the concentration of advanced medical resources in urban areas, coupled with generally higher health awareness among urban residents, contributed to earlier detection and better access to healthcare..

To address these disparities, we recommend a multi-pronged public health strategies for policy-makers to strengthen early screening, intervention, and therapeutic measures to ensure early detection, diagnosis, and treatment for female BC. Additionally, health education and awareness campaigns targeting BC risk factors should be enhanced to promote a healthy lifestyle. Furthermore, medical resources in rural areas should be improved by providing advanced medical equipment and experienced doctors to enhance the BC detection rate. Finally, policy-makers should improve the diagnostic and treatment capabilities of medical staff in primary healthcare institutions to ensure standardized basic medical services for BC. The success of population-based screening depends on the government’s ability to provide high-quality mammography, ensure high coverage and follow-up for screened patients, manage symptomatic patients, and promptly offer diagnostic and treatment services.^[40]^

The mortality rates increased with aging, with the majority of deaths occurring in individuals aged 85 and above, which was consistent with previous studies.^[11]^ Existing studies had shown that females aged 65 and older have the highest mortality rate from BC.^[41]^ Several factors contributed to this elevated mortality in the elderly. Elderly patients usually suffered from underlying diseases or chronic comorbidities,^[42,43]^ and they had a higher likelihood of recurrence and metastasis after treatment, poorer prognosis, and higher mortality rates. Since 2009, The Chinese government had actively implemented and promoted nationwide screening programs for cervical cancer and BC. In 2012, The Chinese government began to carry out BC screening, which was fully launched in 2016. The highest mortality rate among the elderly may be attributed to the positive impact of early BC screening.^[44]^ Timely detection and treatment of lesions had led to improved survival rates and delayed the age of death among female BC patients. However, with the cumulative effect of risk factors over time and the potential for more aggressive tumor biology in later life, mortality rates in the elderly population remained high.^[45]^ Furthermore, studies have demonstrated that 50% of female BC were diagnosed through BC screening programs, while the remaining 50% were diagnosed after the manifestation of symptoms.^[46]^ Elderly female BC may be unable to afford the high treatment costs, which similarly contributed to an increased mortality rate. Additionally, the issues of population aging, low birth rates, and increased life expectancy indirectly contributed to the rise in female BC mortality.^[12]^

In light of these findings, we recommend that special attention should be given to elderly females over the age of 65years. There should be an intensified focus on the dissemination and education of BC prevention and treatment information. It is essential to encourage elderly females to undergo regular medical checkups. This approach will facilitate the early detection, diagnosis, and treatment of BC. Additionally, there should be a heightened emphasis on posttreatment care and rehabilitation. Such a comprehensive approach is essential to improve the quality of life and effectively reduce mortality in this vulnerable population.

Subgroup analyses by age revealed that mortality trends remained relatively stable across the 0 to 24, 25 to 34, 35 to 64, and ≥65-year age groups in the overall females population. However, a further stratification by region uncovered distinct patterns: a statistically significant increasing trend was observed in the 0 to 24-year group in urban areas, and in the 25 to 34, 35 to 64, and ≥65-year groups in rural areas. A critical finding was that although the mortality rate was higher in urban areas, the absolute number of deaths was greater in rural regions. Several interconnected barriers contributed to this disparity. Many rural residents lived far from screening facilities, which increased both time and economic costs and may even lead to patients forgoing screening due to inconvenience. Although screening may be free of charge, the associated costs of diagnosis and treatment creatde a significant financial barrier. Rural areas generally lacked specialized medical facilities and equipment, such as mammography devices, which restricted the accessibility of screening. The number of medical professionals in rural areas, especially radiologists and breast specialists, was limited, which affectted the quality and efficiency of screening. Cultural beliefs in some rural areas may influence women’s acceptance of BC screening, such as feelings of shame or fear about physical examinations. It is necessary to consider various factors, including geography, economy, medical resources, health awareness, and social support, and adopt a multifaceted approach to improve early screening and diagnosis, thereby enhancing the prognosis of rural BC patients.

To our knowledge, this is the first study to analyze changes in the mortality trend of female BC in China using real-world surveillance data from 2014 to 2021. The primary strength of the current study lies in the extraction of comprehensive age-specific mortality data for female BC from the DSP system, covering the period from 2004 to 2021, which ensures a representative sample of the national population. Stratified analyses by region and age group, which maximize the utilization of available data. Comparing the simulated acceleration between different subgroups directly enhances a more concise and intuitive interpretation of the results. However, our study still has limitations. First, unlike the GBD study, we were unable to analyze risk factors for BC using surveillance data, which limits the depth of our article’s content. This will be the next step in our team’s efforts. Second, cause-of-death surveillance does not cover all districts or populations in China, leading to underreported and underestimated mortality. Third, due to the lack of specific socioeconomic information and BC incidence rates, conclusions regarding urban-rural disparities are based on informed speculation. Fourth, although the Joinpoint regression model can quantify the AAPC in BC mortality and identify potential inflection points, it intrinsically represents only a piecewise linear approximation of real-world data. In reality, temporal trends in BC mortality are shaped by a complex interplay of multiple determinants, including the broad implementation of screening programmes, continuous advances in therapeutic strategies, reforms in health-insurance coverage, and pronounced shifts in population age structure. These multifactorial interactions frequently generate trajectories that are nonlinear, subject to time-lagged effects, and heterogeneous across subpopulations.

## 5. Conclusion

The study has identified a significant upward trend in the age-specific mortality rate of BC in rural areas compared to urban areas. Despite substantial investment from the Chinese government, the national coverage rate of BC screening remains low, especially in rural areas. Expanding screening coverage in rural areas and improving the accessibility of BC screening and treatment for the elderly can effectively enhance the utilization of health resources.

## Acknowledgments

We would like to acknowledge the language polishing service provided by Home for Researchers (www.home-for-researchers.com), which greatly enhanced the quality of our manuscript.

## Author contributions

**Conceptualization:** Fengxun Ma, Huali Xiong.

**Data curation:** Fengxun Ma, Huali Xiong.

**Formal analysis:** Huali Xiong.

**Funding acquisition:** Huali Xiong.

**Investigation:** Fengxun Ma, Huali Xiong.

**Methodology:** Fengxun Ma, Huali Xiong.

**Project administration:** Fengxun Ma, Huali Xiong.

**Resources:** Fengxun Ma, Huali Xiong.

**Software:** Fengxun Ma, Huali Xiong.

**Supervision:** Fengxun Ma, Huali Xiong.

**Validation:** Fengxun Ma, Huali Xiong.

**Visualization:** Fengxun Ma, Huali Xiong.

**Writing – original draft:** Fengxun Ma, Huali Xiong.

**Writing – review & editing:** Fengxun Ma, Huali Xiong.
